# Letter knowledge in parent–child conversations: differences between families differing in socio-economic status

**DOI:** 10.3389/fpsyg.2014.00632

**Published:** 2014-06-24

**Authors:** Sarah Robins, Dina Ghosh, Nicole Rosales, Rebecca Treiman

**Affiliations:** ^1^Department of Philosophy, University of KansasLawrence, KS, USA; ^2^Department of Philosophy, Washington University in St. LouisSt. Louis, MO, USA

**Keywords:** home literacy environment, parent-child conversations, socioeconomic status (SES), letter knowledge, Preschool children

## Abstract

When formal literacy instruction begins, around the age of 5 or 6, children from families low in socioeconomic status (SES) tend to be less prepared than children from families of higher SES. The goal of our study is to explore one route through which SES may influence children's early literacy skills: informal conversations about letters. The study builds on previous studies (Robins and Treiman, 2009; Robins et al., 2012, 2014) of parent–child conversations that show how U. S. parents and their young children talk about writing and provide preliminary evidence about similarities and differences in parent–child conversations as a function of SES. Focusing on parents and children aged three to five, we conducted five separate analyses of these conversations, asking whether and how family SES influences the previously established patterns. Although we found talk about letters in both upper and lower SES families, there were differences in the nature of these conversations. The proportion of letter talk utterances that were questions was lower in lower SES families and, of all the letter names that lower SES families talked about, more of them were uttered in isolation rather than in sequences. Lower SES families were especially likely to associate letters with the child's name, and they placed more emphasis on sequences in alphabetic order. We found no SES differences in the factors that influenced use of particular letter names (monograms), but there were SES differences in two-letter sequences (digrams). Focusing on the alphabet and on associations between the child's name and the letters within it may help to interest the child in literacy activities, but they many not be very informative about the relationship between letters and words in general. Understanding the patterns in parent–child conversations about letters is an important first step for exploring their contribution to children's early literacy skills and school readiness.

## Introduction

The early years of formal schooling are devoted to teaching children how to read and write. There are differences among children in their preparedness for this instruction, with some children entering school with more knowledge about letters and print than others. In particular, children from families low in socioeconomic status (SES) tend to be less well prepared for literacy instruction and to perform less well in school than children from more privileged backgrounds (Duncan et al., 1998; McLoyd, 1998; Arnold and Doctoroff, 2003; Ryan et al., 2006). The goal of our study is to explore one route through which SES may exert its influence on children's early knowledge about letters and writing: informal conversations about letters that occur at home. We provide a detailed description of the talk about letters that occurs between U.S. parents and their preschool children, asking whether and how patterns in this talk differ as a function of family SES.

Previous studies have found some SES differences in the early home literacy environment of U.S. children. Much of this research focuses on books and book reading, showing that children in lower SES households have less exposure to books in the home (Vernon-Feagans et al., 2001; Roberts et al., 2005) and are less likely to be read to by their parents (Feitelson and Goldstein, 1986; Lee and Burkam, 2002). Even when book reading does occur between lower SES parents and children, there are differences in the quality of parent behavior during this activity (Whitehurst and Lonigan, 1998; Phillips and Lonigan, 2009). However, a range of activities—beyond book reading—contribute to the home literacy environment and could contribute, in turn, to children's ability to benefit from the reading instruction that is provided at school. Indeed, Phillips and Lonigan (2009, p. 147) recommended that measures of the home literacy environment be expanded to include “literacy artifacts, functional uses of literacy, verbal references to literacy, library use, parental encouragement and value of reading, parental teaching of skills, child interest, parent modeling of literacy behaviors, parental education, and parental attitudes toward education.” The present study is a response to that request. We select one of those recommended activities—verbal references to literacy—and examine whether it differs across families with different SES backgrounds.

We focus on parent–child conversations about letters, in part because previous studies have shown that activities that promote children's focus on letters improve their early literacy skills (Sénéchal et al., 1998; Evans et al., 2000; Hood et al., 2008; Martini and Sénéchal, 2012). Conversations about letters, which can occur across a range of everyday activities, may be an important means by which such activities have their influence. Although parents and children may talk about letters while reading books, they may also do so during activities that are not directly focused on literacy, for example while making dinner or doing chores.

We are encouraged in this line of inquiry by studies showing that patterns in parent speech influence children's learning in domains outside of literacy. For example, when *three* is used in reference to apples, days of the week, and toys, it can prompt children to search for how these disparate sets are similar, encouraging them to think about numerical equivalence and thereby improving their mathematical knowledge once they arrive at school (Mix et al., 2002; Levine et al., 2011). A further motivation for the present study is that interventions designed to promote talk about letters appear to improve children's understanding of written language. For example, when parents and teachers are trained to include more explicit references to print during literacy-related activities, like book reading and joint writing, children's overall letter knowledge improves (Lovelace and Stewart, 2007; Justice et al., 2008).

General differences in the conversational patterns of families differing in SES prompt us to consider whether these patterns will influence how parents and their young children talk about letters. Previous studies have shown differences in both the quantity and quality of mothers' talk to children as a function of SES (Hart and Risley, 1995; Hoff and Naigles, 2002). For example, higher SES parents are more likely to talk to their children in ways that elicit and encourage conversation from the child, whereas lower SES parents are more likely to speak to their children in ways that are focused on directing behavior (Farran and Haskins, 1980; Heath, 1983).

For the present study, our interest is in whether there are further differences in parent–child conversations as a function of SES, specifically, differences in the kind of information that is provided in talk about letters. To explore this question, we must first establish the nature of these conversational patterns. Studies that describe the role of parent–child conversations in the home literacy environment tend to employ one of two methods. Some studies use questionnaires, asking parents about the frequency of certain conversational topics, such as rhyming and alphabet games (Phillips and Lonigan, 2009), or about their approach to talking with their child (Umek et al., 2005). The parents in such studies report that they engage their young children in conversations about letters and print (Phillips and Lonigan, 2009). Other studies document patterns in parent–child talk about letters more directly, carrying out case studies with a single family or a small number of families (Neumann et al., 2008; Edwards, 2012). Such studies reveal that parents offer informative statements about letters such as *That's the letter M for MILK. The letter M makes a MMM sound* (Neumann et al., 2008) or *Both words purple and pink begin with P; those are both P words* (Edwards, 2012). Although studies using questionnaire methods tend to have large samples, parents' responses may reflect, in part, the behaviors they think they should engage in. Even if parents' responses are honest, they may not be aware of or remember many of the relevant conversations. Case studies provide more detail about the content of conversations, but only for a restricted set of families. Many case studies, like the ones mentioned above, focus on families of high SES, raising questions as to whether these results generalize to families of preschool children more broadly.

In a series of recent studies (Robins and Treiman, 2009; Robins et al., 2012, 2014), we developed a method for describing parent–child conversations that combines the advantages of each of the above approaches and minimizes their respective limitations. To provide a direct and detailed account of the patterns found in literacy-relevant conversations across a wide range of families with preschool age children, we have examined parent–child conversations available in CHILDES: an online repository of spoken language transcripts (MacWhinney, 2000). CHILDES transcripts—most of which were collected, initially, for studies of children's spoken language development—provide an excellent resource for identifying whether and how parents and children talk during everyday activities. Our studies demonstrate that preschool children and their parents talk about letters and writing and that the ways in which they do so change across the preschool years.

Our interest in the present study is to determine whether there are differences in patterns of talk about letters as a function of SES. CHILDES, again, serves as a resource for asking this question. A number of the researchers who submitted transcripts to CHILDES provided information about the SES of their participants, as determined by parent education and income. For example, one set of transcripts came from the Home-School Study of Literacy and Language Development (Dickinson and Tabors, 2001), which focused exclusively on low-income families. Menn and Gleason (1986), in contrast, recruited their participants from “middle-class families in the Boston area,” while other researchers included parent–child conversations from both lower and higher SES families (e.g., Hall et al., 1984). Because the researchers who contributed data to CHILDES differed somewhat in the standards they used for classifying families, our inquiries about SES in CHILDES rely on a general distinction between higher and lower SES that is applicable across corpora. Even using such a basic distinction between demographic groups, there is reason to believe that at least some group differences can be detected. Specifically, a previous exploratory analysis of SES differences found some differences in how parents and children talked about letters and pictures as a function of SES (Robins et al., 2012).

More recently, we have conducted detailed investigations of patterns in talk about letters that occurs between U.S. parents and their preschool age children (Robins et al., 2014). This study showed that parents talked to their young children about letters, questioning them about various features of letters, combining letters into sequences, and associating letters with words. Children, too, talked about letters, displaying in their utterances at least a rudimentary understanding of some aspects of letters. Having identified these patterns in parent–child conversations about letters, we now ask whether the patterns differ as a function of SES. The present study thus extends the analyses presented in Robins et al. (2014), tailoring them to the subset of corpora for which we have information about participant SES. While other studies have focused on differences in the quantity of literacy-related interactions across SES groups (e.g., Vernon-Feagans et al., 2001; Umek et al., 2005), our approach focuses on the quality of these interactions, asking about the nature of these conversational patterns in lower and higher SES families.

Specifically, the present study documents patterns in parent and child talk about letters when children are between the ages of 3;0 (years; months) and 5;0, as found in transcripts of parent–child conversations that are available in CHILDES (MacWhinney, 2000). Our study consists of five separate analyses, each of which explores one conversational pattern. We selected patterns that were previously identified in our studies of CHILDES (Robins et al., 2014) and that we hypothesize may differ as a function of family SES. Analysis 1 explores questions asked about letters. Analysis 2 examines utterances that mention associations between letters and words, and Analysis 3 looks at utterances that feature letters in sequence. In our final two analyses we look at the letter names used in these conversations in greater detail, asking about the frequency with which individual letters (monograms) and two-letter combinations (digrams) are used and whether patterns in their use are predicted by the SES of the speakers.

## Analysis 1: questions

Our first analysis focuses on one highly interactive form of conversation: questions about letters. Previous studies have shown that parents and children sometimes ask questions about letters and print (Yaden et al., 1989), and studies of preschool classrooms have shown that teachers vary the type and complexity of questions posed to students (Massey et al., 2008). Our previous survey of parent–child conversations in CHILDES (Robins et al., 2014) revealed that parents and children asked a number of questions about letters, using these questions to inquire about many features of the letters. Here we ask whether the quality of these questions varies as a function of SES.

### Methods

#### Utterances for analysis

For this and all subsequent analyses, we used the same 12 corpora of parent–child transcripts that were included in the previous SES analyses described in Robins et al. (2012). The previous study by Robins et al. (2012) used these transcripts to compare talk about writing and about drawing; here our focus is on talk about letters. All 12 corpora included conversations recorded at home between U.S. parents and children, although two of them also included sessions that occurred in a laboratory setting. Families were classified as either lower SES or higher SES, using the demographic data made available by the researchers in CHILDES (MacWhinney, 2000). Given the lag between collecting data and making it available on CHILDES, many of the corpora include conversations that took place before 2000. Date of transcription could not be included in the formal analyses, however, because individual corpora differed in how they reported it (e.g., by date of recording or date of publication). We included all transcripts of conversations from these corpora that took place between parents and children between the ages of 3;0 and 5;0.

For Analysis 1, we examined all parent and child utterances, defined as a line in the recorded transcript, which included the word *letter* or a specific letter name (as indicated by an *@l* code in the transcript—e.g., *that's a T@l*). We excluded utterances of *letter* that referred to mailed correspondence. We found utterances that met these criteria in the transcripts from 111 of the 158 parent–child pairs in the 12 corpora. Our searches yielded a total of 3074 utterances—1481 for higher SES families (533 for parents, 948 for children) and 1593 for lower SES families (550 for parents, 1043 for children).

#### Coding

For each statement in the transcript that included the word *letter* or a letter name, we asked whether it was a question. We then distinguished between questions that asked about letter-related skills and those that did not. Non-skill questions included those that mentioned letters while asking about some other topic (e.g., *Do you like your ABC soup?*). Skill questions were in turn coded as either elaborative or basic. Elaborative questions were those that required the respondent to provide letter name or sound, identify a letter shape, complete a sequence, or some combination of these skills. Basic questions required only a yes or no answer. For example, *what is the letter that your name starts with?* was coded as an elaborative question, whereas *Is this an A?* was coded as basic.

For this and the following analyses, a second coder analyzed approximately 5% of the utterances, randomly selected from the full set. Inter-rater agreement was never below 88% for any feature, and the two coders agreed 94% of the time overall. To ensure that this agreement was higher than expected by chance, we calculated the Cohen's κ coefficient for each coding. All κ scores were above 0.75.

#### Statistical analysis

As in Robins et al. (2012) and Robins et al. (2014), analyses were carried out with multilevel models, using the lme4 software package (Bates, 2009). By treating corpus and child as random factors, we were able to determine whether patterns in questions were predicted by variables of interest while statistically controlling for the undue influence of any particular corpus or parent–child pair. We ran two multilevel models for each of the coded features of an utterance, as described above (i.e., whether the utterance as a question, whether the question was a skill question, and whether the skill question was elaborative). The first or non-SES model included the factors of child age (in months) and speaker (child or parent), as well as the interaction of these two factors. The second or SES model included the variables from the first model, as well as SES (higher or lower) and the interaction of SES with the previous variables. Child age was centered in each model. Each model included a random intercept for each child and for each corpus. The two models were statistically compared to determine whether the second accounted for significantly more variance than the first. When the SES model predicted significantly more variance and included a significant main effect or interaction involving SES, we report the results of this model. Otherwise, we report results from the non-SES model.

### Results

The results of our question analyses are displayed in Table 1. Although age was treated as a continuous variable in the analyses, the results are broken down into 2 year-long age groups in Table 1 in order to illustrate the findings. A first statistical analysis was carried out to examine the factors that may help to predict whether an utterance that included a letter name was a question. The SES model performed significantly better than the non-SES model (*p* < 0.001). Overall, 16% (487 of 3074) of all statements including letter names or the word *letter* were questions. The percentage of utterances that included a letter name that were questions was lower for lower SES parents and children than for higher SES parents when children were younger. The percentage of questions increased for lower SES families as children grew older, such that the SES groups showed similar percentages of questions when children were between 4;0 and 5;0. These trends were confirmed by the main effect of SES (*p* < 0.001) and an interaction between age and SES (*p* < 0.001) in the SES model. All other effects were non-significant.

**Table 1:** **Proportion of letter statements in analysis 1 that included different types of questions, by SES and child age**.

	**Letter questions as a proportion of all letter utterances**		**Elaborative letter questions as a proportion of all letter skill questions**	
**Child age**	**Lower SES**	**Higher SES**	**Lower SES**	**Higher SES**
3;0–4;0	0.07	0.18	0.25	0.50
4;0–5;0	0.19	0.18	0.14	0.55
Overall	0.14	0.18	0.17	0.53

Nearly all questions—95% (463 of 487)—were classified as skill questions. An analysis designed to predict whether a question was a skill question showed no significant effects in either the non-SES or SES models, and the SES model did not perform significantly better than the non-SES model (*p* = 0.244).

There were, however, differences between SES groups in the types of skill questions asked. In analyses designed to predict whether a skill question was an elaborative question, the SES model predicted more variance than the non-SES model (*p* < 0.001). Overall, 36% (168 of 463) of skill questions were elaborative, requiring the respondent to say something beyond yes or no in order to answer the question. The percentage of skill questions that were elaborative was higher in parents, 49%, than in children, 27%. Collapsing across parents and children, the percentage of skill questions that were elaborative was substantially larger in higher SES families, 53%, than in lower SES families, 17%. Also, higher SES families tended to ask more elaborative questions at the older child ages, whereas lower SES families tended to ask fewer. These trends are supported by the SES model, which showed an interaction between child age and SES (*p* = 0.005), as well as main effects of age (*p* < 0.001), speaker (*p* = 0.002), and SES (*p* = 0.039).

### Discussion

The results of Analysis 1 confirm previous observations that parents and children sometimes ask questions about print (Yaden et al., 1989; Robins et al., 2014). In the later preschool years, a number of these questions not only mention letters but ask about the features of letters directly. There are differences in this highly interactive form of conversation as a function of family SES. Of the letter names they uttered, lower SES parents and children had a smaller proportion that were in questions, and the questions that they asked tended to require less detailed responses. The overall difference in proportion of questions is consistent with previous studies that suggest there are SES differences in the kinds of conversations parents have with their young children (e.g., Farran and Haskins, 1980; Heath, 1983). The further discovery that lower SES families have a smaller percentage of elaborative questions than higher SES families do tempers our previous finding (Robins et al., 2014) that, across the preschool years, the questions that parents ask their young children change from simple questions such as *Where is the I?* to more complex ones such as *Dog starts with D—what letter comes next?* While some parents do this, a change toward more elaborate questions may not happen equally for all children. Given studies that stress the importance of questions for promoting children's interest in and understanding of letters and print (Justice et al., 2008; Massey et al., 2008), our results suggest that lower SES children may be at a disadvantage by having fewer of these interactions.

## Analysis 2: associations

Even when parents and their preschool children are not asking questions about print, their conversations may still promote young children's knowledge about print if they involve statements about the connections between letters and words. Case studies suggest that parents make such letter–word associations, as when the parent in the Neumann et al. (2008) study said *that's the letter M for MILK*, or when the parent in the Edwards (2012) study said *Both words purple and pink begin with P*. In our previous study using CHILDES (Robins et al., 2014), we found that associations between letters and words were common for both parents and children, but that the types of words used in these associations differed as a function of the child's age. Specifically, parents of younger children focused on associations between letters and proper names, and the majority of these letter–name associations involved the child's name.

A child's own name may serve as an important entry point for directing the child's attention toward letters. Given children's interest in their own names, focusing on this association when children are younger may give them an incentive to learn about the connections between letters and words more broadly (Aram and Levin, 2004; Both-de Vries and Bus, 2010). We asked in Analysis 2 whether there were differences in the relative frequency of associations with the child's name between lower and higher SES families.

### Methods

#### Utterances for analysis

All utterances of individual letter names and all utterances of the word *letter* that came from both parents and the target child were included in this analysis. This yielded a total 6169 utterances of letter names and *letter*—1804 from parents and 4365 from children.

#### Coding

First, we coded each utterance of *letter* or individual letter name for whether it was associated with a word. To qualify as associated, both the letter and the word to which it referred needed to be explicitly stated in the same line of the transcript. For example, *D is for dog* and *This is the first letter of your name* were coded as associated, but *d-o-g* was not. For all of the utterances that involved associations, we distinguished between those that were associated with the child's name and those that were associated with other words. Name associations included statements like *Your name starts with J* and *J is for Jason*. Not all corpora provided the first names of the children involved in the study, so this coding method may not have identified all associations with names.

#### Model

The analyses were carried out on each individual utterance of a letter name. Corpus and child were incorporated into the model as random factors, and non-SES and SES models were compared, as described in Analysis 1.

#### Results

A first analysis was carried out to examine the factors that may help to explain whether a letter was associated with a word. There were no influences of SES, as confirmed by comparison of the SES and non-SES models (*p* = 0.276). Parents and children often associated letters with words throughout the age range studied; approximately 3 out of every 10 utterances (1876 of 6169) of letters were associated with a word. Letters were more likely to be associated with words as the child grew older, and parent utterances of letters were more likely to be associated with words than children's. Further, parents' proportion of letter–word associations remained fairly constant across the 3;0–5;0 age range, whereas children's proportion of letter–word associations increased after age 4;0. The model showed main effects of age (*p* < 0.001) and speaker (*p* < 0.001), as well as an interaction between age and speaker (*p* < 0.001).

Of particular interest were the types of words with which the letters were associated. For this analysis of the proportion of associations that were made with the child's name, the SES model predicted more variance than the non-SES model (*p* < 0.001). Associations of a letter with the child's name constituted 22% of all letter-word associations (409 of 1876). There were SES influences on the proportion of associations that involved the child's name, as Table 2 shows. Specifically, we found a higher proportion of child name associations in lower SES families than in higher SES ones. The focus on associations with the child's name was especially strong for lower SES families at the younger ages. These trends were supported by the SES model, which showed an interaction between child age and SES (*p* < 0.001), and main effects of child age (*p* = 0.042) and SES (*p* < 0.001). Collapsing across SES groups, there was a higher proportion of letter–word associations with the child's name for parents than for children. Parents' relative proportion of these associations decreased across the 3;0–5;0 age range more quickly than children's did, as reflected in the main effect of speaker (*p* = 0.002), which was modified by an interaction between child age and speaker (*p* < 0.001).

**Table 2:** **Proportion of child name associations out of all letter–word associations in analysis 2, by SES, speaker, and child age**.

	**Lower SES**			**Higher SES**		
**Child age**	**Parent**	**Child**	**Overall**	**Parent**	**Child**	**Overall**
3;0–4;0	0.66	0.52	0.59	0.36	0.65	0.47
4;0–5;0	0.23	0.35	0.32	0.05	0.46	0.38
Overall	0.54	0.41	0.46	0.21	0.48	0.40

### Discussion

The results of Analysis 2 confirm our previous finding that parents often talk to their young children about letters as being associated with words (Robins et al., 2014). In the present study, more than a third of parent utterances of letters involved associations between letters and words. For both parents and children, many of these associations featured the child's name. The emphasis on the child's name was particularly strong in lower SES families. That is, while the proportion of letter–word associations was similar for lower and higher SES families, lower SES families were particularly likely to make associations with one particular word: the child's name. While this association may draw the child's interest, serving as a critical starting point for making letter–word associations more broadly, persisting with this particular association may not be highly informative. For example, some studies have suggested that children may treat their own names as special, failing to generalize from this association to others (Drouin and Harmon, 2009).

## Analysis 3: sequences

One important feature of letters is that they come in sequences. Uttering letters in sequence may provide information that letters form a class of symbols. Some sequences of letters, however, are more informative than others. The alphabetic sequence helps children learn the letter names, but the order of this sequence is unrelated to the order with which letters appear in words or to other characteristics of the letters, such as the nature of the sounds that they symbolize. Our previous study (Robins et al., 2014) showed that parents and children often used letters in sequences and that during the later preschool years—which are the focus of the present study—parents and children increasingly focus on the sequences of letters that make up words over those that make up the alphabetic order sequence.

Questionnaires surveying parents about the home literacy environment indicate that many parents consider reciting the alphabet to be an important literacy-related activity and that do this often (e.g., Phillips and Lonigan, 2009). There may, however, be differences among families in the extent and duration of this focus on alphabetic order. In our previous analyses of SES effects in CHILDES (Robins et al., 2012), we found preliminary indications that lower SES families emphasized the alphabetic sequence more than higher SES families. Lower SES families were, for example, more likely to talk about the letters A, B, and C as belonging to children (e.g., asking *do you know your ABCs?*). As the Robins et al. (2012) study focused on comparisons between writing and drawing, the possibility of SES differences in letter sequences was not explored further in those analyses. In the present analysis, we asked which utterances of letter names were made as part of a sequence, and further, what kinds of sequences were used. We asked whether lower and high SES families differed in these regards.

### Methods

#### Utterances for analysis

This analysis included only uses of individual letter names, leaving out use of the word *letter*. There were yielded 5899 uses of individual letter names—1654 for parents and 4245 for children.

#### Coding

Each letter name was first coded for whether it was part of a sequence. A sequence was defined as any instance of two or more symbols in a row, where symbols could be either letters or numbers, separated at most by *and*. For example the letter names in the utterances *2L, AB and C, XX42J*, and *D-O-G* would all be coded as being in a sequence, whereas the letters in the utterances *I put A on top of B* and *I see two Ds* would not. Then, we asked about the length of each sequence, counting each letter name or number as a token. Then, for all of the letters that were in a sequence, we asked whether the sequence was in alphabetic order. All sequences featuring consecutive letters of the alphabetic order sequence met this criterion, even if they began in the middle of the alphabet (e.g., *lmnop*).

#### Model

The analyses of letter sequences were carried out on individual uses of letter names. Corpus and child were incorporated into the model as random factors, as in Analyses 1 and 2.

### Results

In an analysis designed to predict whether a letter occurred in a sequence, the SES model predicted more variance than the non-SES model (*p* < 0.001). Letters were more likely to be uttered in sequence than not—65% (3813 of 5899) of all letter names were said as part of a sequence. The proportion of letter names in sequence for the different groups is shown in Table 3. The children had more sequences than parents and, while children's proportion of sequences remained relatively constant across the 3;0–5;0 age range, parents' proportion of sequence utterances increased at the older child ages. Collapsing across parents and children, there were also SES differences in the frequency of sequence utterances. Higher SES families had a higher proportion of letter sequence utterances than lower SES families, and this was especially due to the relatively small proportion of sequences for lower SES families from 3;0–4;0. These results are supported by main effects of child age (*p* = 0.025), speaker (*p* < 0.001), and SES (*p* = 0.020) in the SES model, as well as interactions between child age and speaker (*p* < 0.001) and child age and SES (*p* < 0.001).

**Table 3:** **Proportion of letter names that occurred in sequences in analysis 3, by SES, speaker, and child age**.

	**Lower SES**			**Higher SES**		
**Child age**	**Parent**	**Child**	**Overall**	**Parent**	**Child**	**Overall**
3;0–4;0	0.38	0.66	0.58	0.51	0.72	0.66
4;0–5;0	0.61	0.67	0.65	0.64	0.71	0.69
Overall	0.50	0.66	0.62	0.58	0.71	0.68

Overall, 42% (1212 of 2918) of children's sequences were in alphabetic order, whereas only 27% (239 of 895) of parent sequences were in alphabetic order. There were influences of SES on the proportion of sequences that were in alphabetic order, as reflected in the better performance of the SES model relative to the non-SES model (*p* < 0.001). These results are displayed in Table 4. Lower SES families had a higher proportion of alphabetic order sequences than higher SES families, and this effect was particularly due to the use of such sequences after age 4;0. That is, while higher SES families showed a decline in the proportion of sequences that were in alphabetic order at the older child ages, lower SES families did not. These trends were supported by the SES model, which showed main effects of speaker (*p* = 0.004) and SES (*p* < 0.001), modified by an interaction between child age and SES (*p* < 0.001).

**Table 4:** **Proportion of letter sequences that are alphabetic order sequences in analysis 3, by SES and child age**.

**Child age**	**Lower SES**	**Higher SES**
3;0–4;0	0.48	0.56
4;0–5;0	0.36	0.20
Overall	0.41	0.35

There were no differences across speaker or SES in the length of sequences that were uttered, and the SES model did not perform better than the non-SES model in predicting sequence length (*p* = 0.233). Overall, the average sequence length was 4.43 letters. Sequences tended to be shorter at the older child ages, shrinking from an average length of 4.79 from 3;0–4;0 to 4.20 from 4;0–5;0, as supported by a main effect of age (*p* = 0.015). The shortening of sequences and the lack of a difference between parents and children, while initially surprising, may reflect changes in the type of sequence uttered across the 3;0–5;0 age range. At the younger ages, many of the sequences uttered were alphabetic order sequences, and children had a higher relative proportion of alphabetic order sequences than parents.

### Discussion

Analysis 3 confirms our previous findings (Robins et al., 2014) that U.S. parents and their young children often use letters in sequences and that many of these sequences feature letters in alphabetic order. The present study also extends and refines those results, showing an influence of SES on both letter sequence utterances. While all parents used sequences of letters, lower SES parents uttered a lower proportion of letters in sequences than did higher SES parents and more letters individually. Moreover, of the letter sequences that parents used, lower SES parents had a higher proportion of alphabetic order sequences, especially when their children were older than 4 years of age. Our finding that lower SES families place more emphasis on memorizing the alphabet in order supports and extends the results of previous studies (Baker et al., 1998; Robins et al., 2012).

Learning the alphabetic sequence is enjoyable for young children, particularly when it is done through songs. It may help to draw children's attention to letters, promoting an interest in learning to read and write. But learning how to read and write requires an understanding of how letters combine to form words, and this is not information that can be gleaned from memorizing letter names in alphabetic order. The fact that lower SES children hear many alphabetic sequences, even during the later preschool years, suggests that they may be at an informational disadvantage relative to higher SES children.

## Analysis 4: monograms

The previous three analyses establish SES differences in the questions that parents and children ask about letters, the types of associations they make between letters and words, and how they combine letters into sequences. Having established these differences between SES groups, we use the present analysis to step back and ask a more basic question: Are there differences in the individual letters that are used? Using a method developed in our previous study of parent–child letter talk (Robins et al., 2014), we asked whether some individual letter names (monograms) are used more often than others and whether these differences in frequency of use reflect various features of a letter, such as its position in the alphabet and frequency in English words. In our initial study, we found that parents and children often used the letters *A, B*, and *C*, but that with older children the frequency of letters used increasingly reflected the frequency with which individual letters occur in English words. We build on that earlier analysis in Analysis 4, asking whether these general patterns in frequency of monogram use differ as a function of SES.

### Methods

#### Utterances for analysis

This analysis used the same 5899 uses of individual letter names identified in Analysis 3.

#### Coding and analysis procedure

Letter name utterances were pooled into 2 year-long age groups: 3;0–4;0 and 4;0–5;0. We ran separate regression analyses to predict the number of utterances of each letter name by parents and by children. The dependent variable was the frequency of each letter's use, log transformed in order to make the distribution more normal. The predictor variables included child age group, SES, position in the alphabet, and frequency in words. Our position measure, which we label ABC, distinguished the three letters at the beginning of the alphabet—the ones that are often used to label the sequence—from the remaining 23 letters. The frequency variable reflects how often particular letters occur in English words. It was measured here as the number of occurrences of the letter across the 6231 words that appear in Zeno et al. (1995) survey of written materials for kindergarten and first-grade children. Because this variable showed moderate positive skew, we performed a square root transformation. In addition to the variables of child age, ABC, and letter frequency, the analyses included the interactions between child age and ABC and between child age and letter frequency. All continuous variables were centered in the analyses.

### Results

#### Parents

There were no SES influences on parent monogram utterances, nor did SES interact with any of the other variables. For parent utterances we found a significant effect of ABC (*p* < 0.001). Parents used the letter names A, B, and C significantly more often than expected on the basis of other factors. Indeed, 24% (394 of 1654) of parents' monogram utterances were one of these three letters. Parents' rate of A, B, and C utterances did not differ across the age range studied, as indicated by the lack of an interaction between the child age and ABC variables. Parent monogram utterances were also predicted by the frequency of these monograms in English words (*p* < 0.001), and this variable also did not interact significantly with child age.

#### Children

There was no effect of SES on children's monogram utterances. The results for children's monogram utterances were similar to those for parents: we found significant effects of ABC (*p* < 0.001) and the frequency of the letter in English words (*p* < 0.001). The first three letters of the alphabet constituted 22% (954 of 4.245) of children's monogram utterances throughout the 3;0–5;0 age range. The emphasis on A, B, and C continued across this period, as indicated by the lack of an interaction between child age and ABC. The frequency of the letter in English words was also a significant predictor of children's monogram use, and there were no interactions with child age.

### Discussion

The letters that U.S. parents and children most often talk about are those at the beginning of the alphabet, which are the ones that are often used as a label for the alphabet sequence, and those that frequently occur in English words. These results align with those of our previous study of monogram use (Robins et al., 2014). Given that the present study examines a subset of the corpora used in that initial analysis, the similarity of these findings is to be expected. What is of interest is that we found no differences in the frequency of monogram use as a function of SES. Given that Analyses 1–3 demonstrate several ways in which the patterns of letter talk differ between higher and lower SES families, one might have suspected that these differences would extend to basic letter use as well. While families of different SES backgrounds differ in the questions that they ask about letters, the ways in which they associate letters with words, and the sequences of letters that they use, there were no effects of SES—as a main effect or interaction—for parents or for children in the individual letters that they used.

## Analysis 5: digrams

Analysis 3 revealed differences in the sequences of letters that parents and children use as a function of SES, but Analysis 4 revealed no influence of SES on the factors that influence use of individual letter names. For our final analysis, we explore the possibility of SES differences in the factors that influence the frequency of letter use at an intermediate level—two-letter combinations, or digrams. Our previous study of parent–child letter use (Robins et al., 2014) revealed that digrams can serve as an informative unit of analysis. In that study we found that parents and children used some pairs of letters more often than others and, further, that these differences reflected properties of the digrams themselves, above and beyond properties of the individual letters within them. Here we take that inquiry a step further, asking whether there are SES differences in the letters that parents and children combine into basic sequences.

### Methods

#### Coding and analysis procedure

For Analysis 5, we used the set of letter name sequences identified in Analysis 3 and identified each digram in the sequence. For example, if a child said *D-O-G*, the utterance was coded as involving two digrams, *D-O* and *O-G*. The 26 letters of the alphabet can be combined to create 676 distinct digrams, and we kept track of how often each digram occurred in parent and child speech for each year group. The transcripts analyzed contained 2960 digrams—671 from parents and 2289 from children.

Our analysis of the factors that influence parent and child digram use includes 12 factors. First, we used the monogram variables from Analysis 4, applied separately to each letter in the digram (Letter 1, Letter 2): child age, SES, Letter 1 ABC, Letter 2 ABC, Letter 1 frequency, and Letter 2 frequency. We then added the set of digram-level variables used in Robins et al. (2014): digram ABC, digram alphabet, digram frequency, and digram repeat. The digram ABC variable distinguished the digrams involved in the ABC sequence—*A-B* and *B-C*—from the remaining 674 digrams. The digram alphabet variable coded each digram for whether the two letters were in alphabetic order, as in *A-B, J-K*, and *X-Y*. Digram frequency was calculated using the same set of words from children's books used to analyze monograms in this and the previous analysis. Finally, the digram repeat variable distinguished between digrams that repeated the same letter (e.g., *J-J* and *P-P*) and those that did not (e.g., *E-F, B-L*).

### Results

#### Parents

The results of the regression analyses for parent digram utterances are shown in Table 5. Parent digram utterances were influenced by a range of factors, many of which echo the findings of Robins et al. (2014). First, parent utterances of two-letter sequences were influenced by several features of the individual letters in those sequences, including the frequency of each letter in English words (Letter 1: *p* < 0.05, Letter 2: *p* < 0.01) and whether the first letter of the digram was A, B, or C (*p* < 0.05). Parents showed a tendency to use digrams that were in alphabetic order (*p* < 0.001), and of the alphabetic order digrams, parents were most likely to utter A-B or B-C (*p* < 0.001). Further, parents' digram use was predicted by repetition (*p* < 0.01) and by the frequency of the digram in English words (*p* < 0.001). There was, in addition, a main effect of age (*p* < 0.001), reflecting the presence of more letters uttered in combination after child age 4.

**Table 5:** **Summary of regression analyses for predictors of child and parent digram utterances in analysis 5**.

	**Child**		**Parent**	
**Predictor**	***B***	***SE B***	***B***	***SE B***
SES	0.027	0.120	−0.014	0.082
Child age	0.114^***^	0.024	0.065^***^	0.016
× SES	0.002	0.034	0.014	0.023
**LETTER 1 PREDICTORS**				
Letter ABC	0.105^**^	0.038	0.067^*^	0.026
× SES	−0.007	0.054	0.026	0.037
Frequency	0.0005^***^	0.00009	0.0001^*^	0.00006
× SES	−0.00007	0.0001	−0.00004	0.00009
**LETTER 2 PREDICTORS**				
Letter ABC	0.066	0.038	0.044	0.026
× SES	−0.070	0.054	−0.009	0.037
Frequency	0.0005^***^	0.0001	0.0002^**^	0.00007
× SES	0.00003	0.0001	−0.00005	0.00009
**DIGRAM PREDICTORS**				
Letter ABC	1.382^***^	0.235	1.215^***^	0.162
× SES	−0.263	0.332	−0.059	0.229
Frequency	0.021^***^	0.004	0.016^***^	0.003
× SES	0.012	0.006	0.012^**^	0.004
Alphabetic order	1.781^***^	0.066	0.832^***^	0.046
× SES	0.098	0.094	−0.586^***^	0.064
Repeat	0.701^***^	0.062	0.125^**^	0.042
× SES	−0.225^*^	0.088	−0.020	0.060

N = number of utterances. Overall model for children, F_(19, 2684)_ = 143, p < 0.001, R^2^ = 0.500. Overall model for parents, F_(19, 2684)_ = 57.99, p < 0.001, R^2^ = 0.286.

* p < 0.05.

** p < 0.01.

*** p < 0.001.

SES alone did not predict the digram use for parents, but it did interact with some of the other variables. There was an interaction between SES and digram frequency (*p* < 0.01). Higher SES parents were more likely than lower SES parents to utter digrams that reflected pairs of letters found in English words. There was also an interaction of SES and alphabetic order digrams (*p* < 0.001), but this trend was in the opposite direction: parents from lower SES families were more likely than parents from high SES families to utter alphabetic digrams.

#### Children

The results of children's digram utterances are also displayed in Table 5. There were several monogram-level influences on children's digram utterances, echoing the patterns identified in Robins et al. (2014)—the frequency of each letter in words of the language (Letter 1: *p* < 0.001, Letter 2: *p* < 0.001) and whether the first letter of the digram was *A, B*, or *C* (*p* < 0.01). At the digram level, there were main effects of each variable: frequency (*p* < 0.001), digram ABC (*p* < 0.001), alphabetic order (*p* < 0.001), and repeat (*p* < 0.001). Many of children's digram utterances involved the sequences at the beginning of the alphabet. Children's digram utterances were also significantly predicted by the child's age (*p* < 0.001), with more digram utterances for children after age 4.

There was no main effect of SES on children's digram use. SES did, however, interact with another variable of interest: digram repeat (*p* < 0.05). This interaction qualifies the main effect of repeated digrams identified above. Children were significantly more likely to utter the same letter twice than would have been expected on the basis of other factors, and this was especially true for lower SES children.

### Discussion

The results of this final analysis provide further insight into SES differences in talk about letters. Although there are no differences among parents and children of higher and lower SES backgrounds in the factors that influenced their use of individual letter names, there are SES differences in how they put letters together. Most of the differences we identified came from the parents' use of letters. Although many of the parents we studied used sequences of letters in their conversations with their young children, the kinds of sequences they used differed as a function of SES. Lower SES parents had a higher proportion of alphabetic order sequences. In contrast, higher SES parents showed a stronger tendency to use sequences of letters that that are common in words of the language. This marks an important difference in the input children receive about how letters go together.

We identified only one influence of SES on children's digram use: lower SES children were more likely than their higher SES peers to repeat the same letter. While letters are occasionally doubled in the spelling of words, this tendency to repeatedly say a single letter name may reflect a focus on naming letters rather than combining them to spell words.

## General discussion

There are well-established differences in children's preparedness for reading and writing instruction at the beginning of formal schooling as a function of SES. Children from lower SES backgrounds arrive at school with less understanding of letters and how they can be combined to form words, and this gap only widens over the subsequent years (Duncan et al., 1998; Arnold and Doctoroff, 2003; Ryan et al., 2006). There is thus a strong interest in understanding the nature of the child's home environment prior to formal schooling and in identifying factors that may contribute to these differences. Previous studies have found general differences in the conversational patterns of parents and children as a function of SES (e.g., Hart and Risley, 1995; Hoff and Naigles, 2002), as well as differences in the quantity and quality of literacy-related activities such as book reading (Vernon-Feagans et al., 2001; Roberts et al., 2005). The present study examined a feature of the home literacy environment at the intersection of these two activities: parent–child conversations about letters.

The present study builds on a prior investigation of parent–child conversations about letters in CHILDES (Robins et al., 2014) which identified five general patterns in these conversations: questions about letters, associations between letters and words, types of letter sequences, as well as the frequency with which individual letters (monograms) and two letter sequences are used. In that previous study, we found that parents and their young children asked questions about letters, made associations between letters and words, and used letters in sequences, although the kinds of questions, associations, and sequences changed across the preschool years. In the current study, we used demographic information about the parent–child pairs—made available by the researchers who submitted their transcripts to CHILDES—to explore whether these previously established patterns differed as a function of SES. We found some important influences of SES on the previously established patterns. While parents and children in both higher and lower SES groups talk about letters during everyday activities, there are SES differences in the features of these conversations that influence how engaging and informative the interactions are for young children.

One way that parents can engage their young children is by asking them questions. Questions are considered a highly interactive form of conversation (Massey et al., 2008), and we found differences in the prevalence of this form of conversation as a function of family SES. When lower SES parents and children talked about letters, a smaller proportion of those utterances were questions than was the case for higher SES parents and children. Further, of the questions lower SES families asked, a lower proportion required a detailed response. By using a lower proportion of their utterances about letters to query their children about the letters' features, lower SES parents may do less to draw their children's attention to print in environment. This, in turn, may lead children to be less inquisitive about the letters and words that they see printed on such things as toys, signs, and food boxes. Higher SES parents appear to take better advantage of impromptu opportunities to incorporate information about letters into everyday activities.

Parents can, of course, engage their children in other ways, for example by focusing on topics of interest to the child. With regards to reading and writing, two such topics are the alphabetic sequence and the child's name. Singing the alphabet song and writing or orally spelling the child's name are enjoyable activities that may help to motivate children to attend to print (Aram and Levin, 2004; Both-de Vries and Bus, 2010). Our study shows that both activities occur in the parent–child pairs we examined. Although these activities are valuable, understanding how letters function to produce words requires going beyond these initial activities to discuss sequences other than the alphabet and words other than the child's name. We found SES differences in how parents extend their discussions of letter sequences and associations. Lower SES parents appear to persist in the focus on the alphabetic sequence and simple associations between the child's name and letters of the alphabet for longer than their higher SES counterparts. So, although both high and low SES children receive information about sets of letters and connections between letters and words, the information they can glean from these conversations differs. Higher SES children appear to have more opportunities to learn about how letters can combine to form a range of words. Our findings are consistent with those of previous studies in which lower SES mothers report believing that helping children with basic letter-related skills is important (Fitzgerald et al., 1991; DeBaryshe, 1995). Lower SES parents may be getting the message that it is important to teach their young children about letters, but they may need further guidance on the range of content these interactions should include.

Documenting features of the home literacy environment and how they vary across families is important because it can suggest routes via which we could intervene to improve the literacy outcomes for lower SES children. As ours was a descriptive study, and because it did not follow children as they entered school, we are unable to draw conclusions about the relationship between the conversational patterns we identified and the later literacy achievements of this specific group of children. A further limitation of our approach is that we could only make a brute distinction between higher and lower SES families. Although we found significant differences between these two groups, we encourage further studies that explore finer-grained distinctions between SES groups and seek to disentangle the various demographic factors that contribute to SES classification. Moreover, because recording and transcribing conversations for inclusion in CHILDES takes time, there is the potential for a gap between the patterns observed and current home literacy practices. Nonetheless, we are confident that our study offers important insight into how parent–child conversations can be studied and into the nature of those conversations. By taking advantage of the information available in CHILDES, we were able to examine a much larger sample of conversations, everyday activities, and families than we could have otherwise. Our sample was larger than that of most previous studies of the home literacy environment, and our analyses more detailed. Our study provides an important and previously unavailable baseline for further studies that explore the nature of the home literacy environment in the U. S. and other countries, how it varies across families, and how it influences children's progress when formal literacy instruction begins at school.

### Conflict of interest statement

The authors declare that the research was conducted in the absence of any commercial or financial relationships that could be construed as a potential conflict of interest.
